# Editorial: Delivering nucleic acids to immune and non-immune cells

**DOI:** 10.3389/fimmu.2023.1237506

**Published:** 2023-08-08

**Authors:** Kirill A. Afonin, Francesca Re, Diana Boraschi

**Affiliations:** ^1^ Nanoscale Science Program, Department of Chemistry, University of North Carolina Charlotte, Charlotte, NC, United States; ^2^ Department of Medicine and Surgery, University of Milano Bicocca, Milano, Italy; ^3^ Shenzhen Institute of Advanced Technology (SIAT) of the Chinese Academy of Science (CAS), and China-Italy Joint Laboratory of Pharmacobiotechnology for Medical Immunomodulation, Shenzhen, China; ^4^ Institute of Biochemistry and Cell Biology, Consiglio Nazionale delle Ricerche, Napoli, Italy; ^5^ Stazione Zoologica Anton Dohrn, Napoli, Italy

**Keywords:** delivery, nucleic acids, vaccination, antigen presentation, nanoparticles, NANPs

For decades, there has been ongoing research focused on the targeted delivery of therapeutic DNA and/or RNA molecules designed to regulate the expression of specific genes. This field of study aims to advance new clinical approaches for therapies that target the genetic components of various diseases in a personalized manner. The technology had an impressively rapid advancement and gained global recognition, particularly in the new vaccination strategies adopted during the COVID-19 pandemic (1, 2). However, a major obstacle that still hinders the broader success of nucleic acid-based therapies and vaccines lies in the inefficient delivery of these biopolymers to human cells, as well as in ensuring their subsequent intracellular release and optimal performance. The challenges arise from the inherent limitations of naked, non-modified oligonucleotides, among which are their susceptibility to rapid degradation by nucleases and renal clearance, the inefficient crossing of biological membranes, and the potential to induce uncontrolled immunological responses (3, 4). To optimize the efficacy while minimizing the harmful side effects of nucleic acid cargos, recent research has focused on improving the design of delivery systems suitable for rationally designed DNA and RNA molecules (5). A special emphasis on targeting immune cells is warranted in the case of vaccination. The strategy aims to shuttle the antigen-coding nucleic acids to cells that will be able to synthesize, process, and present the antigens. By doing so, optimally protective immune responses will lead to the development of long-term protective immunological memory.

The current Research Topic features a collection of fifteen review and research articles curated by international leaders in the fields of nucleic acid therapies, immunology, and drug delivery. The manuscripts present a range of innovative technologies encompassing the design of various nucleic acid-based therapeutics, assessment of their biological activities, and optimization of administration conditions.

Antigen-encoding nucleic acids can be optimized for a particular application through several strategies. The review by Vishweshwaraiah and Dokholyan provides a comprehensive assessment of rational design and optimization strategies for mRNA vaccines, highlights different platforms available for vaccine delivery, and discusses the limitations and future challenges associated with this emerging technology.

By encoding appropriate target proteins, such as the Spike protein of SARS-CoV-2, and selecting between the B- and T-cell epitopes, it becomes possible to elicit either antibody or cellular responses. Also, the specification of CD4 and CD8 epitopes can assist in the stimulation of helper and cytotoxic responses, respectively. The research by Del Riego et al. provides an analysis of the levels of SARS-CoV-2 specific antibodies and T cells in intensive care unit workers. The aim is to gain additional insights into their immune protection following vaccination. Additionally, Becker et al. share longitudinal vaccination response data in dialysis patients and control subjects, as part of a mixed mRNA vaccination scheme, and Raptis et al. report differences in immune responses for patients with inflammatory rheumatic diseases, which depended on the type of administered mRNA vaccine. Garcia-Dominguez et al. discover that the dosing intervals in mRNA vaccination could improve the durability of immune responses, with longer intervals being preferable.

The inclusion of chemical modifications and sequence optimization plays a crucial role in defining the stability, cellular localization, and therapeutic efficacy of delivered mRNA (6, 7). These strategies enable fine-tuning mRNA physicochemical properties, enhancing its lifetime, and optimizing its performance as a therapeutic agent. In their research article, Bai et al. present a nucleoside-modified Rabies mRNA-lipid nanoparticle vaccine able to induce prolonged immune responses in animal models, while Rice at al. employ heterologous vaccination to elicit robust cellular immunogenicity with increased protection against the emerging variants of SARS-CoV-2 infection.

Nucleic acid technologies, combined with the innate capacity of the human immune system to detect nucleic acids and initiate efficient immune/inflammatory responses, have paved the way for their potential use as adjuvants and immunostimulants (8). These applications are crucial for enhancing vaccine efficacy. The review by Gu et al. provides a good overview on the relationship between circular RNA and immunity, highlighting the current applications and future directions of these technologies. Consequently, it is imperative to focus research efforts on the design of nucleic acids that can induce controlled inflammation in a limited manner, both temporally and anatomically. For instance, this can be achieved by targeting Toll-like receptors (TLRs) or NOD-like receptors (NLRs). A clinical trial reported by Daniel et al. reveals the first-in-human Phase 1 study of immunostimulatory spherical nucleic acids designed to target TLR9. The results make this compound promising as an immunotherapy agent.

Recent advancements in nucleic acid bioengineering have led to the emergence of nucleic nanoparticles (NANPs), multistranded nanoassemblies composed exclusively of RNA, DNA, and their chemical analogs, which offer an innovative approach for regulated personalized immunostimulation and drug delivery (9, 10). By changing the composition and architectural features of NANPs, it becomes possible to target the activation of specific pattern recognition receptors (e.g., TLR7 or RIG-I) and promote the delivery of scaffolded antigens and therapeutics to the diseased cells (11, 12). Panigaj et al. review recent developments and applications of NANPs, rationally designed for therapeutic immunomodulation, and identified current limitations and future directions of this innovative platform.

Efficient intracellular delivery of nucleic acid therapeutics relies on the use of various carriers, among which are viral vectors, highly efficient but with problems of immunogenicity and specificity of targeting, lipid nanoparticles (LNPs), currently the lead delivery systems for nucleic acid-based vaccines and therapeutics, exosomes, extracellular vesicles, a recent promising delivery and diagnostic technology that is still at early stages of its development, as well as various inorganic nanoparticles and polymers (13, 14). In their review, González-Rioja et al. revise the synthetic methods, physicochemical characterization, and pharmacokinetics of surfactant ionizable lipid nanoparticles loaded with RNA therapeutics. The review articles by Gao et al. and Zhang et al. discuss the application of extracellular vesicles and exosomes as new diagnostic tool and drug delivery carriers for cancer immunotherapies, respectively. Finally, Gusta et al. develop a panel of cationic gold nanoparticle-based nanovectors used for the safe and sustained internalization of mRNAs via endocytosis. The obtained results with cell culture experiments show promise for this technology to be used as a delivery platform for nucleic acids.

With further developments of nucleic acid-based technologies, it is essential to address the potential risks associated not only with therapeutic cargos but also with other formulation components that can lead to excessive inflammation, cytokine storms, and the development of autoimmune and inflammatory diseases (15, 16). The review by Dobrovolskaia shares the valuable insights obtained from the extensive experience of the Nanotechnology Characterization Laboratory regarding the interactions between different nanoparticles and the immune system, which significantly impact the safety and effectiveness of formulations.

In summary, while nucleic acid therapies hold immense promise and have achieved significant advancements, their broader applications still necessitate further improvements, among which are increasing delivery efficiencies, providing storage and handling of all formulations at ambient temperatures, design-driven regulation of immunorecognition and toxicities, and lowering production costs by addressing technological hurdles and logistical challenges (17).

## Author contributions

KA, DB, and FR contributed to writing and revision of the manuscript. All authors contributed to the article and approved the submitted version.
